# Modulation of ACE2/Ang1-7/Mas and ACE/AngII/AT1 axes affects anticancer properties of sertraline in MCF-7 breast cancer cells

**DOI:** 10.1016/j.bbrep.2024.101738

**Published:** 2024-05-23

**Authors:** Reihaneh Fatehi, Mohammad Nouraei, Morteza Panahiyan, Marzieh Rashedinia, Negar Firouzabadi

**Affiliations:** a Student Research Comittee, Shiraz University of Medical Sciences, Shiraz, Iran; b Department of Pharmacology & Toxicology, School of Pharmacy, Shiraz University of Medical Sciences, Shiraz, Iran; c Food and Supplements Research Center, Shiraz University of Medical Sciences, Shiraz, Iran; d Medicinal Plants Processing Research Center, Shiraz University of Medical Sciences, Shiraz, Iran

**Keywords:** Renin-angiotensin system, Breast cancer, Sertraline, Losartan, AT1 receptor, Mas receptor, Angiotensin 1-7

## Abstract

The renin–angiotensin system (RAS) is best known for playing a major role in maintaining the physiology of the cardiovascular system. Dysregulation of the RAS pathway has been proposed as a link to some malignancies and contributes to cancer metastasis.

Breast cancer is considered as one of the leading causes of cancer death in women and its prevention remains yet a challenge. Elements of RAS are expressed in both normal breast tissue and cancerous cells, signifying the essential role of RAS in breast cancer pathology. Sertraline, a widely used antidepressant, has shown anti-proliferative properties on a variety of malignancies.

This study aimed to investigate the effect of sertraline and its combination with agonists and antagonists of RAS (A779, Ang 1–7 and losartan) on viability of MCF-7 cells along with their effect on apoptosis and distribution of cell cycle. Our results indicated that sertraline, losartan and Ang 1–7 significantly decreased cell viability, induced apoptosis and cell cycle arrest. A779 blunted the effect of sertraline on cell viability, ROS generation and cell cycle arrest. Combination treatment of sertraline with losartan as well as Ang 1–7 caused a remarkable decline in cell viability.

In conclusion, results of the present study support the anti-cancer properties of sertraline, losartan and Ang 1–7 via induction of apoptosis and cell cycle arrest.

## Highlights

• Sertraline, losartan and Ang 1-7 decreased MCF-7 cell viability, induced apoptosis and cell cycle arrest.
• A779 blunted the effect of sertraline on cell viability, ROS generation and cell cycle arrest.
• Combination of sertraline and losartan decreased MCF-7 cell viability.
• Combination of sertraline and Ang 1-7 decreased MCF-7 cell viability.
• Anticancer properties of sertraline in MCF-7 cells could be mediated by Mas receptors.

## 1 Introduction

The renin–angiotensin system (RAS) plays a major part in the physiology of the cardiovascular system [1]. RAS mediates its physiological and pathological effects by means of two pathways: ACE/Ang II/AT1R and ACE2/Ang1-7/MasR. Stimulation of ACE/Ang II/AT1R leads to vascular constriction as well as cell proliferation. The second axis is usually responsible for opposite actions [2]. Multiple studies have proposed dysregulation of the RAS pathway as a direct link to some malignancies such as prostate, ovarian, and breast cancers [[3], [4], [5]]. Dysregulation of local RAS subsidizes to cell proliferation, angiogenesis and cancer metastasis [6].

Breast cancer is considered as one of the leading causes of cancer death in women worldwide [7]. Based on previous reports, all RAS elements are expressed both in normal breast tissue and in cancer cells, suggesting the important role of RAS in breast cancer pathology [3]. A recent study showed that in normal breast tissue, the elements of ACE-2/Ang1–7/Mas receptor pathway were predominantly expressed, while in breast cancer tissue, the ACE/Ang II/AT1 receptor pathway was activated prominently [8]. In breast cancer, by activation of Ang II-AT1 axis plentiful intracellular kinases like Akt and MAPK are triggered which will lead to cell proliferation, angiogenesis, migration, inflammation, and apoptosis inhibition [9]. AT1R activity has been shown to be linked with the development and metastasis of breast cancer. Hence, targeting this axis by means of AT1R antagonists such as losartan may serve as an anti-breast cancer. It has been shown that AT1R blockers such as losartan preclude the oncogenic properties provoked by AT1R signaling [10,11].

Angiotensin 1–7 (Ang1–7) is one of the bioactive elements of RAS, which is produced by the enzymatic reaction of angiotensin-converting enzyme 2 (ACE2) on the cleavage of Ang II [12]. A779 is a selective Mas receptor antagonist that exhibits no significant affinity for AT1 and AT2 receptors. Ang1-7 plays anti-proliferative, anti-angiogenic, and anti-fibrotic roles in various cancers by binding to Mas receptors [13,14]. Previous studies have shown that cancer cell growth was inhibited by Ang 1–7 [15,16]. In addition, Ang II-induced angiogenesis and metastasis was impeded by Ang1-7 and by means of inhibiting the expression of VEGF and MMP-9 [17]. Ang 1–7 counteracts the effect of Ang II in breast cancer cells. As observed, Ang 1–7 expression is down-regulated in breast cancer and continues to decline with cancer progression [18]. These results indicate that inhibition of the Ang II-AT1 axis or stimulation of the Ang 1-7-Mas axis could assist in controlling the progression of cancer. In this regard, RAS could be proposed as a therapeutic goal for treatment of breast cancer.

Sertraline, a selective serotonin reuptake inhibitor, is vastly utilized in patients with depression. The effect of RAS genetics on response to sertraline was previously reported [19]. In recent years, sertraline has gained attention owing to its anti-proliferative effects on a various types of malignancies [[20], [21], [22], [23], [24]].

In this study the potential antineoplastic properties of sertraline and Ang 1–7 along with their action when used in combination with A779, an antagonist of Mas receptors, on MCF-7 cancer cells were studied. Additionally their potential effects on apoptosis as well as their effect on cell cycle distribution were assessed.

## 2 Materials and methods

### 2.1 Materials

Sertraline and losartan were obtained from Exir pharmaceutical company, Tehran, Iran. Ang 1–7 and A779 (Ang1-7/Mas receptor antagonist) were obtained from Tocris Bioscience, Bristol, UK. For all experiments, Ang1-7 and A779 were used at a final concentrations of 10^−6^ M (1 μM). Concentrations were selected based on a pilot study. MCF-7 cells were pre-treated with A779 (1 μM) for 2h before exposure to 1 μM Ang 1–7 and 25 μM sertraline.

### 2.2 Cell line and culture

Breast carcinoma cell line MCF-7 was obtained from Pasteur Institute, Iran. Cells were cultured in Dulbecco's modified Eagle's medium (DMEM) (Bioidea, Iran) supplemented with 10 % fetal bovine serum (FBS) (Gibco, USA) and incubated at 37 °C with 5 % CO2.

### 2.3 Cell viability assay

Effect of sertraline, losartan, Ang 1–7, A779 alone and in combination on cell viability was evaluated using 3-(4, 5-Dimethylthiazol-2-yl)-2, 5-Diphenyltetrazolium Bromide (MTT) (Sigma-Aldrich, St. Louis, MO, USA). MCF-7 cells were seeded in a 96-well plate (5 × 10^3^ cells/well) overnight to allow adherence and treated with sertraline (25 μM), Ang1-7(1 μM), losartan (50 μM), and A779 (1 μM) alone and in combination for 24hrs. Subsequently, cells were cultured with 10 μl of MTT solution (5 mg/mL) for an additional 2h at 37 °C. Absorbance was measured at 570 nm with an ELISA reader (BD, USA).

### 2.4 Detection of ROS

To measure intracellular ROS level in MCF-7-treated cells, cells were washed with PBS and incubated with 10 μM 2′-7′dichlorofluorescin diacetate (DCFH-DA) (Sigma-Aldrich,St. Louis, MO, USA) at 37 °C for 30 min in the dark. Thereafter, the cells were rewashed with PBS and then lysed with 90 % DMSO/10 % PBS for 10 min at room temperature and in the dark. ROS generation (fluorescence intensity) was then measured using a FLUOstar Omega® fluorimeter (λ excitation=448 nm and λ emission=525 nm) [25].

### 2.5 Cell cycle distribution

MCF-7 cells were seeded and treated as for MTT assay for 24hrs. After treatment, cells were harvested and fixed with 70 % ice-cold ethanol for 2h at −20 °C. Afterwards, cells were incubated with 100 μg/ml propidium iodide (PI) (Sigma-Aldrich, St. Louis, MO, USA) and 100 μg/ml RNase A (Yekta Tajhiz Azma, Tehran Iran) for 30 min at room temperature in the dark, as previously described [26]. Fluorescence of 15,000 cells was measured using a flow cytometer (FACS Calibur) to determine cell cycle distribution.

### 2.6 Apoptosis assay

Cell apoptosis was measured using an Annexin V-FITC/PE Apoptosis kit (Zist pazhouhan, Iran) according to the manufacturer's instructions. MCF-7 cells were collected and re-suspended in 500 μL of binding buffer (1X). Afterwards, stained with Annexin-V- fluorescein isothiocyanate (FITC) and PI for 15 min at 37 °C in the dark. Finally, percentage of apoptotic population in samples were detected by flow cytometry (FACS Calibur) and Flowjo software version 10.5.3.

### 2.7 Statistical analysis

GraphPad prism software package version 8.0.2.263 for Windows, GraphPad Software, La Jolla California USA, www.graphpad.com was used for data analysis. All Continuous variables are demonstrated as mean ± SD. One-way ANOVA test was used to analyze parametric data. Bonferroni multiple comparisons was used as the post-hoc test. Kruskal–Wallis and Dunn's as the post-hoc multiple comparison test was used for non-parametric data. P-value <0.05 was considered as statistically significant.

## 3 Results

MTT assay was carried out to determine the cytotoxicity of different concentrations of losartan, sertraline, Ang [[1], [2], [3], [4], [5], [6], [7]] and A779 on MCF-7 cells.

**Fig. 1:**
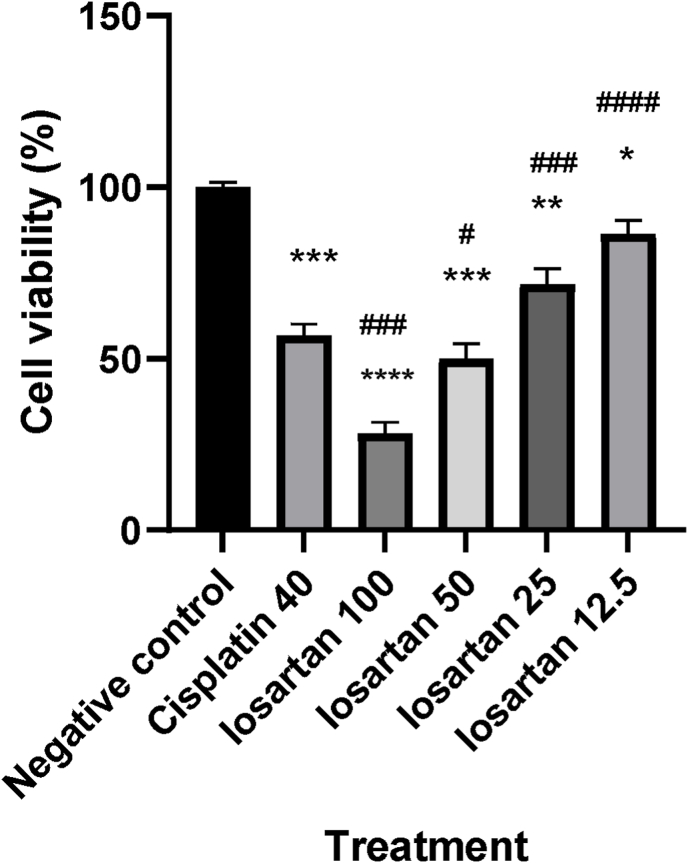
Effect of different concentrations of losartan (100, 50, 25 and 12.5 μM) on MCF-7 cell viability. Each bar is presented as mean ± SD of at least three independent tests. *: P < 0.05, **: P < 0.01, ***: P < 0.001, and ****: P < 0.0001 compared to control and #: P < 0.5, ##: P < 0.01, ###: P < 0.001 and ####P < 0.0001 compared to cisplatin.

**Fig. 2:**
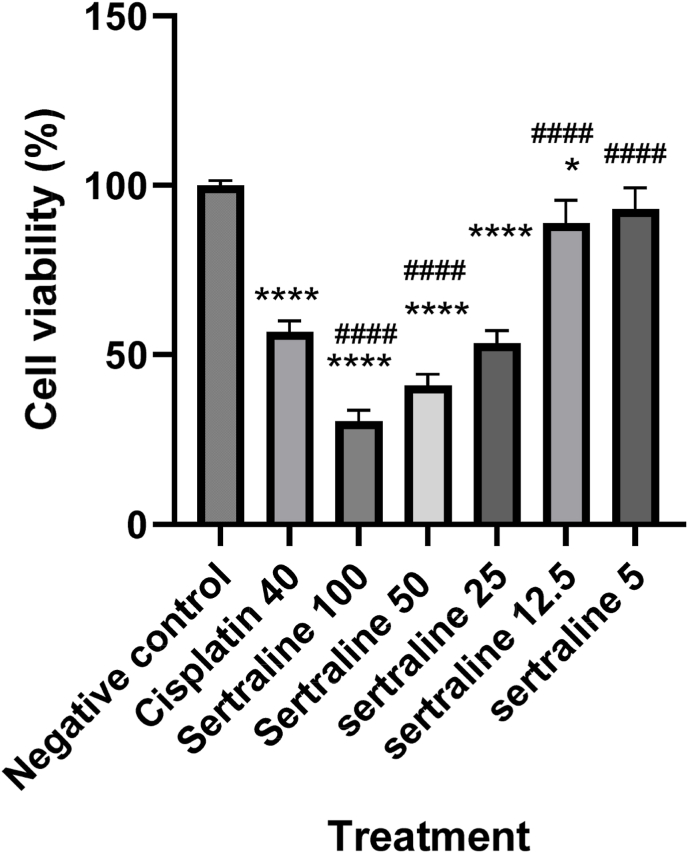
Effect of different concentrations of sertraline (100, 50, 25, 12.5 and 5 μM) on MCF-7 cell viability. Each bar is presented as mean ± SD of at least three independent tests. *: P < 0.05, **: P < 0.01, ***: P < 0.001, and ****: P < 0.0001 compared to control and #: P < 0.5, ##: P < 0.01, ###: P < 0.001 and ####P < 0.0001 compared to cisplatin.

**Fig. 3:**
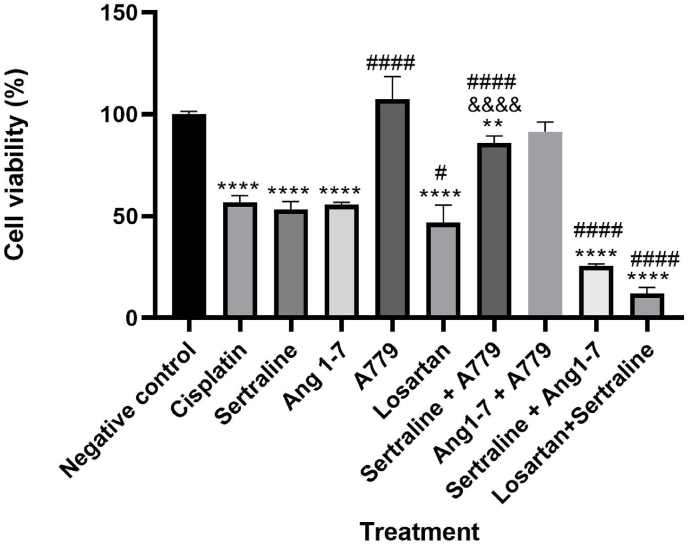
Effect of sertraline, Ang1-7, losartan, and their combination on MCF-7 cell viability. Cells were treated with sertraline (25 μM) in presence of A779 (1 μM) and in combination with Ang1-7(1 μM) and losartan (50 μM) for 24 h. Cell viability was compared to the control. Each bar is presented as mean ± SD of at least three independent tests. *p < 0.05, **p < 0.01, ***p < 0.001, and ****p < 0.0001 compared with negative control. ^&&&&^P < 0.0001 compared with sertraline group. #: P < 0.5, ##: P < 0.01, ###: P < 0.001 and ####P < 0.0001 compared to cisplatin.

As shown in Fig. 1, cell proliferation decreased with increasing concentrations of losartan, with 50 % inhibitory concentration of 50 μM (P < 0.0001). The inhibitory effect of losartan 50 μM was comparable with cisplatin as the positive control (P > 0.05). As shown in Fig. 2, cell proliferation decreased with increasing concentrations of sertraline, with 50 % inhibitory concentration of 25 μM (P < 0.0001). The inhibitory effect of sertraline 25 μM was comparable with cisplatin as the positive control (P > 0.05). Additionally, as shown in Fig. 3, combination of losartan and sertraline significantly inhibited MCF-7 cell growth (P < 0.001). The inhibitory effects of Ang1-7, alone and in combination with sertraline on viability of MCF-7 cells were investigated. The inhibitory effect of sertraline as well as Ang 1–7 was comparable with cisplatin as the positive control (P < 0.05). Sertraline combined with Ang1-7 could significantly increase inhibition in the viability of MCF-7 cells (up to 50 %), compared to treatment with sertraline or Ang1-7 alone (P < 0.0001) (Fig. 3).

A779, a selective Mas receptor antagonist, was administrated 2h before angiotensin1-7 and sertraline treatment. Pretreatment with A779 partially inhibited the effect of sertraline treatment (P < 0.01). MTT assay further demonstrated that incubation of MCF-7 cells alone with A779 exerted no significant effect on cell viability compared to control group (P > 0.05). As presented in Fig. 3, the anticancer effect of Ang1-7 which could inhibit MCF-7 cell viability (P < 0.0001), was blunted by pretreatment with A779.

**Fig. 4:**
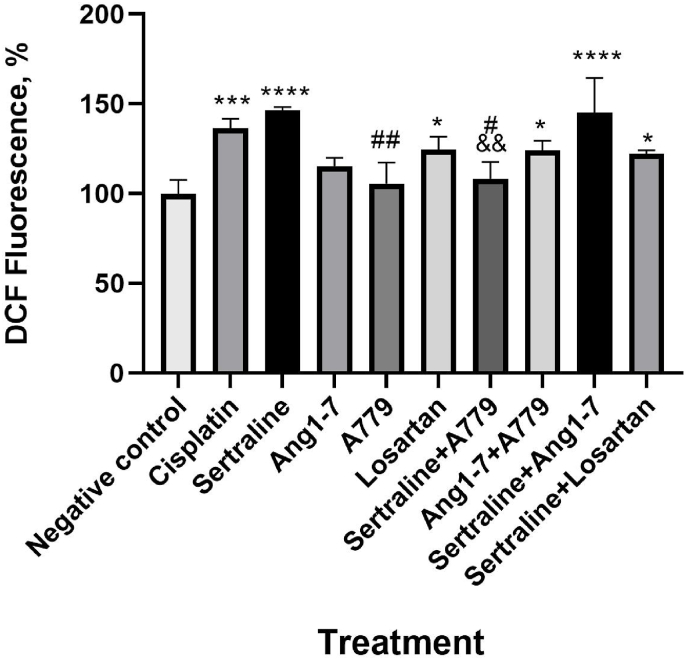
Effects of sertraline, Ang1-7, losartan and their combination on cellular ROS in MCF-7 cells. Cellular ROS production was detected by DCFH-DA staining. Cells were treated with sertraline (25 μM) in presence of A779 (1 μM) and in combination with Ang1-7 (1 μM) and losartan (50 μM) for 24 h. Corresponding quantitative analysis of ROS levels are shown. Each bar is presented as mean ± SD of at least three independent tests *p < 0.05, **p < 0.01, ***p < 0.001, and ****p < 0.0001compared with negative control.

Regarding ROS generation, losartan significantly increased ROS production in MCF-7 cells compared to the untreated group (P < 0.05). As shown in Fig. 4, sertraline treatment for 24 h, induced ROS generation compared to control group (P < 0.0001). Additionally, ROS generation in both of these groups (losartan and sertraline) was comparable with Cisplatin (P=0.841 and P=0.947, respectively). ROS generation in the combination treatment of losartan and sertraline was significantly higher than that of the control group (P=0.024). There was no significant difference compared to Cisplatin (P > 0.05). Intracellular ROS levels in MCF-7 in response to Ang1-7 treatment did not change significantly (P=0.053). Co-treatment of Ang 1–7 and sertraline was associated with higher ROS production compared with the control group (P < 0.0001) which was comparable with cisplatin (P=0.975). Pretreatment of cells with A779 did not affect ROS production which was comparable to the effect of cisplatin as positive control (P=0.991). As shown in Fig. 4, A779 alone showed no effect in ROS generation in MCF-7 cells compared to negative control (P=0.997) and significantly different compared to Cisplatin (P=0.013). Sertraline-induced ROS generation was modulated when pre-treated with A779. ROS generation in Ang 1–7 cells pretreated with A779 was not significantly different with Ang 1–7 group (P=0.192).

**Fig. 5:**
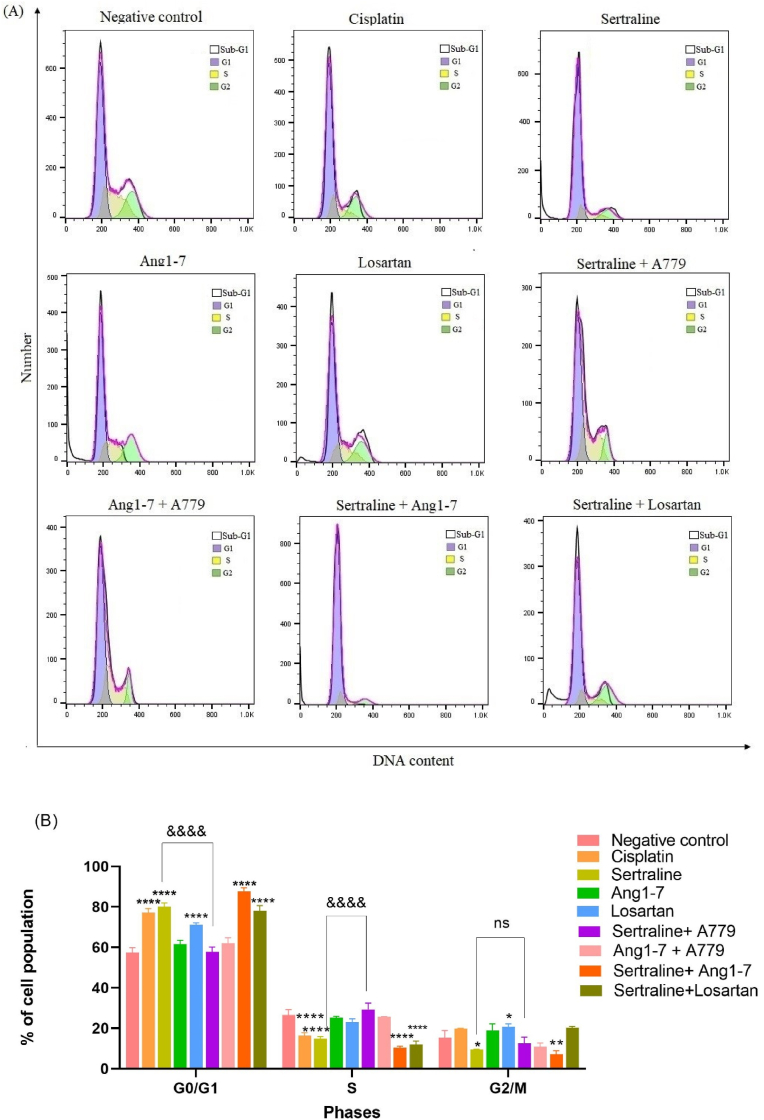
Effects of sertraline, Ang1-7, losartan, and their combination on cell cycle distribution. (A) MCF-7 cells were treated with sertraline (25 μM) in presence of A779 (1 μM) and in combination with Ang1-7 (1 μM) and losartan (50 μM) for 24 h followed by staining with propidium iodide and analysis by flow cytometry. (B) Percentage of cell count in each cycle after being treated with indicated concentrations of sertraline, Ang1-7, losartan, and their combination was calculated. Each bar represents the mean ± SD of three independent experiments. *p < 0.05, **p < 0.01, ***p < 0.001, and ****p < 0.0001 compared with negative control. ^&&&&^P < 0.0001 compared with sertraline group.

To further assess the cytotoxic effects of losartan, MCF-7 cells were cultured with 50 μM losartan and flow cytometry analysis was performed to determine cell cycle distribution and apoptosis. As presented in Fig. 5A and B, losartan inhibited MCF-7 cell progression by increasing percentage of G0/G1 and G2/M phase's population from 57.6 % to 71.3 % and 19.1 %–21.6 %, respectively (P < 0.0001, P = 0.043, respectively). The increase of MCF-7 cell population in both phases was along with a decline of cell count in the S phase; although statistically insignificant. A sub-G1 peak was observed, indicating the apoptotic cells. Moreover, MCF-7 cells treated with sertraline for 24 h exhibited a significant increase in G0/G1 phase arrested (P < 0.0001) and a decrease in cell count in both S and G2/M phases (P < 0.0001 and P=0.02 respectively). In response to sertraline treatment, cell count in G0/G1 phase significantly increased from 57.6 % to 78.3 %, and the count in S-phase declined from 29.6 % to 14 %. In addition, sertraline induced a sub-G1 peak. Furthermore, cell count in G0/G1 phase cells was significantly reduced from 78.3 % to 59.2 % when sertraline treatment was accompanied with A779 pretreatment for 24 h (P < 0.0001). Ang1-7 treatment did not alter the population of cells in G0/G1, S, and G2/M phases compared to untreated group. Meanwhile, the cell cycle distribution pattern through Ang1-7 treatment was approximately similar to that of the untreated group. Cell cycle distribution in Ang 1–7 pretreated with A779 was not statistically different with the control group, as well (P > 0.05).

Furthermore, sertraline and Ang1-7 co-treatment caused a much more cell cycle arrest in G0/G1 than either of the treatments. This treatment showed higher G0/G1 population compared to control group (P < 0.0001). These results suggested that sertraline and Ang1-7 co-treatment led to cell cycle arrest in the G0/G1 phase. Furthermore, sub-G1 peak was observed after MCF-7 cells were treated with this combination. Combination treatment of sertraline and losartan exhibited a significant increase in G0/G1 phase from 57.6 % to 75 % and a decrease in S phase from 29.6 % to 13 % (P < 0.0001). Sub-G1 peak could also be seen in this treated group.

**Fig. 6:**
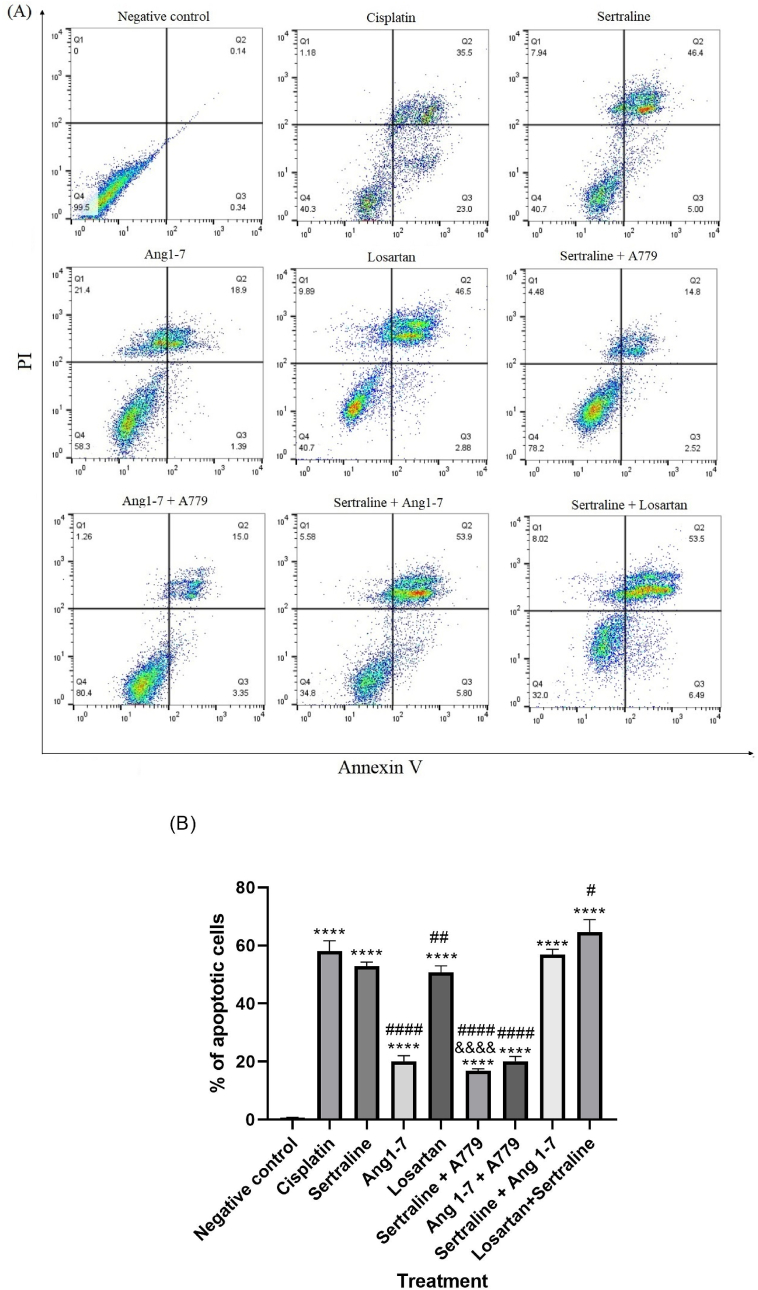
Effects of sertraline, Ang1-7, losartan, and their combination on apoptosis. MCF-7 cells were treated with sertraline (25 μM) in presence of A779 (1 μM) and in combination with Ang1-7(1 μM) and losartan (50 μM) for 24 h to examine their effects on apoptosis. (A) Apoptotic cells were counted Flow cytometry (B) Percentage of apoptotic cells (Q1: necrosis, Q2: late apoptosis, Q3: early apoptosis and Q4: live cells). Each bar represents mean ± SD of three independent tests. *p < 0.05, **p < 0.01, ***p < 0.001, and ****p < 0.0001 compared with negative control. ^&&&&^P < 0.0001 compared with sertraline group. #: P < 0.5, ##: P < 0.01, ###: P < 0.001 and ####P < 0.0001 compared to cisplatin.

Regarding apoptosis, treatment with losartan or sertraline alone, significantly increased apoptotic cells comparing with the untreated group (P < 0.0001) which was comparable with Cisplatin (P=0.620 and P=0.078, respectively). Combination of losartan and sertraline also significantly increased apoptosis compared with control group as well as cisplatin group (P < 0.0001 and P=0.0175respectively). As shown in Fig. 6A and B the apoptotic breast cancer cells were increased in Ang1-7 treatment compared with the control group (P < 0.0001). The rate of apoptosis was 20.29 %. Notably, Ang1-7 treatment caused marked necrotic cell death (21.4 %), as well Increase in apoptotic cell death was also observed in the combination of sertraline and Ang1-7 compared with control group (P < 0.0001). In comparison with sertraline alone, pre-treatment with A779 inhibited sertraline-induced apoptosis from 51.4 % to 17.32 % (P < 0.0001), As presented in Fig. 6A and B, while pre-treatment with A779 exhibited no significant difference in apoptosis compared with Ang1-7 alone, necrotic cell death was significantly reduced. Based on our results, percentage of live cells were markedly increased (80.4 %).

## 4 Discussion

This study provides evidences regarding anticancer potential of sertraline and losartan in human breast cancer MCF-7 cells through ROS generation and induction of oxidative stress. Alongside, both drugs, sertraline and losartan, affected apoptosis and cell cycle distribution in almost the same manner as cisplatin. Our results were suggestive of a possible interaction of sertraline with Mas receptors involved in sertraline-induced apoptosis and cell cycle arrest.

Sertraline is considered as an anti-depressant drug to treat depression and anxiety in cancer patients. In addition to its anti-depressive activity, sertraline exhibits anti-cancer properties in various cancer cells including acute myeloid leukemia, breast, and melanoma cancer cells [20,27,28]. Dysregulation of RAS components has been documented in breast cancer. A recent study indicated that the production of Ang 1–7 was markedly decreased in breast cancer cells, whereas the expression of Mas receptor was upregulated [8]. Other RAS components, in particular, AT1R, are overexpressed in human breast cancer tissue, as well [29]. Activation of Mas receptor and its downstream pathways counteract with the proliferative effects of ACE/AngII/AT1 axis [8]. Therefore, substances with Mas receptor agonist activity appear to offer new opportunities in breast cancer therapy. Our study explored how sertraline and Ang1-7 individually or in combination targeted breast tumor growth. Sertraline treatment caused a significant decrease in cell viability of breast cancer MCF-7 cell. The results were in line with previous reports advocating the anti-proliferative effect of sertraline in various cancer cells [23,30,31]. In our study, the growth inhibitory effect of sertraline in cultured MCF-7 cells were partially blocked by A779 suggesting that the inhibitory effect of sertraline might in part be mediated by Mas receptors. Moreover, our data revealed that Mas activation by Ang1-7 inhibited the viability of MCF-7 breast cancer cells. This result is consistent with previous findings in which Ang1-7 was considered as a potent inhibitor of cell proliferation in lung, breast, nasopharyngeal carcinoma, and hepatocellular cancers [[32], [33], [34], [35]]. Furthermore, we demonstrated that the concurrent treatment of sertraline and Ang1-7 resulted in augmented growth inhibition in MCF-7 cells.

A previous study revealed that over expression of AT1R in breast cancer cells could accelerate tumor growth and increase tumor angiogenesis and invasiveness [17]. Several epidemiological studies have reported that use of AT1 receptor blockers (ARBs) markedly improved patients’ outcomes and survival rates in multiple types of malignancies [36,37]. ARBs could also be plausible treatment options for breast cancer. The current study indicated that losartan alone suppressed the proliferative effects stimulated by AT1. Furthermore, when combined with sertraline, losartan displayed an enhanced anti proliferative effect, caused by apoptosis induction, as proved by flow cytometry analysis. Therefore, we suggested that application of an antagonist of AT1 receptors and an SSRI like sertraline simultaneously may result in suppression of MCF-7 cells proliferation.

ROS are produced as a byproduct of oxygen metabolism that regulates diverse cellular functions, such as cell proliferation and differentiation. Excessive generation of ROS could be considered as a therapeutic target due to its ability to induce oxidative stress and promote apoptosis in cancer cells. Some early studies have shown that sertraline was aberrantly able to augment the accumulation of ROS in cells, while other studies reported that sertraline could inhibit ROS generation [[38], [39], [40]]. These controversial results may indicate that sertraline influences ROS production via distinct signaling networks in different cell lines. Based on our findings it is proposed that sertraline may act as a pro-oxidant in MCF-7 cells. Pretreatment of cells (treated with sertraline) with a Mas receptor antagonist, A779, showed that the stimulatory effect of sertraline on ROS production was abolished by the A779, demonstrating that Mas activation is involved in sertraline-mediated ROS generation in MCF-7 cells. In fact, it may be proposed that the antidepressant sertraline could affect the local RAS in breast cancer cells by interacting with the Mas receptors. However, complementary experiments such as Western blot and immunohistochemistry are needed to further confirm this finding. Moreover, Ang1-7 was not capable of increasing ROS level as previously reported [41,42]. However, co-treatment of Ang 1–7 and sertraline significantly increased ROS production. These observations confirmed that the stimulatory effect on ROS production was largely attributed to sertraline. Additionally, we found an increase in the ROS levels in Ang1-7 group pretreated with A779, which is suggestive of the fact that A779 blunted the antioxidant effect of Ang 1–7 in MCF-7 cells.

Cell cycle dysregulation and apoptosis evasion are considered as hallmarks of cancerous cells. A recent study demonstrated that sertraline causes G0/G1 arrest in prostate stem cancer cells [21]. Sertraline-induced G0/G1 phase arrest was also observed in gastric and colon cancer cells [43,44]. In agreement with earlier studies, the obtained results from flow cytometry indicated that sertraline-treated cells were arrested at G0/G1phase of cell cycle, which caused delayed transition to S phase. Therefore, disruption of cell cycle progression may be proposed as one of the possible underlying mechanisms by which sertraline exhibits its anti-proliferative effects. Furthermore, it was observed in our study that inhibition of the Mas receptor by A779 may lead to significant reduction in the proportion of cells at G0/G1 phase of cell cycle and enhance proliferation of MCF-7 in the groups treated with sertraline which may suggest the role of Mas receptors in anticancer properties of sertraline. The current study provides novel evidences indicating sertraline treatment could stimulate G0/G1 cell cycle arrest via Mas receptor in breast cancer cells. Interestingly, our data showed that Ang1-7 has induced apoptosis, but no significant alteration in cell cycle was observed. Furthermore, our results illustrated that addition of Ang1-7 to sertraline-treated cells, amplify the anti-proliferative effect of sertraline caused by G0/G1 cell cycle arrest. G0/G1-phase arrest of cell cycle proceeds to undergo repair mechanisms or activate apoptosis in cancer cells. Previous studies have shown that sertraline induced apoptosis in various cancer cells such as osteosarcoma, hepatocellular, and lung [38,45,46]. Consistent with previous observations, the present study illustrated that sertraline alone promoted breast cancer cells cytotoxicity via induction of apoptosis. Moreover, co-treatment with Ang1-7 peptide led to an increase in apoptotic cell population. Similar findings have been previously observed in hepatocellular carcinoma, demonstrating Ang1-7-induced apoptosis was caused by an increase in caspase 3 activity [33]. In the current study, it was observed that sertraline-induced apoptosis was markedly attenuated by pre-treatment with A779 in cultured breast cancer cells, suggesting that activation of Mas receptor by sertraline is contributed to the induction of apoptosis and cell growth inhibition.

Increased levels of ROS play a part in the activation of apoptosis cascade. Contribution of ROS to sertraline-induced apoptosis was confirmed by the prior studies. Since our results showed that sertraline induced a substantial increase in the intracellular ROS level and oxidative stress, we hypothesized that sertraline-induced apoptosis is partially dependent on excessive ROS accumulation, which may be mediated through Mas receptors. It is worth noting that the apoptosis rates in MCF-7 cells were less than the cell death detected by MTT test. Earlier studies reported that sertraline induced autophagy-related cell death in prostate and non –small cell lung cancer cells [21,47]. Therefore, it may be postulated that autophagy-mediated cell death might be responsible for this fraction of cell death. Regarding anti-cancer effect of losartan, some in vitro studies on nasopharyngeal, colorectal, and liver cancer have shown that losartan suppresses tumor growth and promotes apoptosis [[48], [49], [50]]. In line with previous studies, our results illustrated that the anti-tumor activity of losartan may be mediated by overproduction of ROS and oxidative stress, leading to increased apoptosis.

## 5 Conclusion

Results of the present study support the anti-cancer properties of sertraline, losartan and Ang 1–7 via induction of apoptosis, cell cycle arrest and ROS generation. Markedly, it may be proposed that anticancer properties of sertraline in MCF-7 cells could be mediated by Mas receptors and that patients with breast cancer may benefit from addition of somewhat safe drugs such as sertraline and losartan to conventional anticancer therapies.

Further investigation of the role of ACE2/Ang1-7/Mas and ACE/AngII/AT1 pathways on anticancer effects of sertraline is warranted.

## CRediT authorship contribution statement

**Reihaneh Fatehi:** Writing – review & editing, Writing – original draft, Data curation. **Mohammad Nouraei:** Writing – review & editing, Writing – original draft, Methodology, Investigation, Formal analysis, Data curation. **Morteza Panahiyan:** Writing – review & editing, Writing – original draft, Methodology, Investigation, Data curation. **Marzieh Rashedinia:** Writing – review & editing, Writing – original draft, Methodology. **Negar Firouzabadi:** Writing – review & editing, Writing – original draft, Supervision, Software, Project administration, Methodology, Funding acquisition, Formal analysis, Data curation, Conceptualization.

## Declaration of competing interest

The authors declare the following financial interests/personal relationships which may be considered as potential competing interests: Dr. Negar Firouzabadi reports financial support was provided by 10.13039/501100004320Shiraz University of Medical Sciences. Dr. Negar Firouzabadi reports a relationship with 10.13039/501100004320Shiraz University of Medical Sciences that includes: funding grants. None declared. If there are other authors, they declare that they have no known competing financial interests or personal relationships that could have appeared to influence the work reported in this paper.

## Data Availability

Data will be made available on request.
